# Clinical observation on the treatment of ankle fracture with buttress plate and traditional internal fixation and its effect on GQOLI-74 score and Baird-Jackson score

**DOI:** 10.12669/pjms.39.2.6876

**Published:** 2023

**Authors:** Zhen Lin, Lian-yun Gao, Kang-ming Ruan, Deng-bing Guo, Yan-hua Chen, Qing-ping Liu

**Affiliations:** 1 Zhen Lin, Department of Trauma, Pediatric Orthopaedics, Mindong Hospital Affiliated to Fujian Medical University, Fu’an 355000, Fujian, China; 2 Lian-yun Gao, Department of Trauma, Pediatric Orthopaedics, Mindong Hospital Affiliated to Fujian Medical University, Fu’an 355000, Fujian, China; 3 Kang-ming Ruan, Department of Trauma, Pediatric Orthopaedics, Mindong Hospital Affiliated to Fujian Medical University, Fu’an 355000, Fujian, China; 4 Deng-bing Guo, Department of Trauma, Pediatric Orthopaedics, Mindong Hospital Affiliated to Fujian Medical University, Fu’an 355000, Fujian, China; 5 Yan-hua Chen, Department of Trauma, Pediatric Orthopaedics, Mindong Hospital Affiliated to Fujian Medical University, Fu’an 355000, Fujian, China; 6 Qing-ping Liu, Department of Trauma, Pediatric Orthopaedics, Mindong Hospital Affiliated to Fujian Medical University, Fu’an 355000, Fujian, China

**Keywords:** Buttress plate, Traditional internal fixation, Clinical observation

## Abstract

**Objective:**

To explore the curative effect of buttress plate and traditional internal fixation in the treatment of ankle fracture, so as to provide potential reference for the clinical treatment of this disease.

**Methods:**

This is a clinical comparative study. The subjects of this study were one hundred patients with ankle fracture treated in Mindong Hospital Affiliated to Fujian Medical University from January 2019 to December 2021. Enrolled patients were randomly divided into the control group and the experimental group. Patients in the control group were treated with traditional internal fixation, and those in the experimental group were provided with buttress plate. Patients were compared in several aspects such as the comprehensive quality of life assessment questionnaire (GQOLI-74), Baird-Jackson score and postoperative complications.

**Result:**

The experimental group showed improved Baird-Jackson score after treatment, significantly higher fracture healing rate than that of the control group three months after treatment. Besides, there was no significant difference in the complications between the two groups, with good prognosis after timely treatment.

**Conclusion:**

Internal fixation with buttress plate has obvious advantages in the treatment of ankle fractures, which can effectively improve the quality of life and promote the rapid healing of fractures. It is worthy of clinical promotion and application.

## INTRODUCTION

Ankle fracture, as an intra-articular fracture, is a common fracture in clinical treatment.^1^ It occurs frequently in young adults, and its incidence rate accounts for 3.9% of the total incidence of fractures. The approach to restore the anatomy of the normal joint is a common choice for treatment, with the purpose to ensure the stability of early movement.^2^ It is generally believed that the normal integration of tibia and fibula is an important reason to ensure the relative stability of human ankle joint.^3^ The posterior ankle is also the end point of the posterior tibiofibular syndesmosis ligament. The posterior ligament in the structure of the distal tibiofibular syndesmosis complex can be responsible for 42% of the overall stability function.

Therefore, the integrity of the posterior ankle structure and function can ensure the normal ankle joint.^4^ In most cases, there is no tear of the posterior tibiofibular syndesmosis ligament when there is a posterior malleolus fracture. However, posterior ankle is the insertion point of the posterior ligament, consequently, fracture at this site will also affect the stability of distal tibiofibular syndesmosis.^2^ According to previous studies^5^, fixation of the posterior malleolus restored the stability of distal tibiofibular syndesmosis to 70%, but screw insertion to distal tibiofibular syndesmosis provided only 40% stability.

Therefore, surgery is commonly performed when the posterior malleolus fracture involves >25% of the distal tibial articular surface. With respect to the above, there are few clinical cases about the selection of specific fixation for posterior malleolus fracture in the clinical treatment. Among them, the method of open reduction and internal fixation is a common therapeutic choice for ankle joint^6^, which, however, has disadvantages of large surgical incision and high risk of postoperative complications. Internal fixation with buttress plate is a new treatment scheme. It has been reported that cannulated screw has a good effect on maintaining the biomechanical strength of posterior malleolus fracture, which can exert an anatomical reduction effect. Therefore, a prospective controlled study was performed to explore the curative effect of buttress plate and traditional internal fixation in the treatment of ankle fracture, so as to provide potential reference for the treatment of this disease.

## METHODS

This is a clinical comparative study. On hundred patients with ankle fracture treated in Mindong Hospital Affiliated to Fujian Medical University from January 2019 to December 2021 were included in the study. Enrolled patients were randomly divided into the control group and the experimental group, with 50 cases in each group. The experimental group had 14 males and 36 females, with an average of 65.9+2.1 years (50-71 years); in terms of the causes of injury, there were 18 cases of traffic accident injury, 19 cases of falling injury and 13 cases of sprain. While there were 13 males and 37 females in the control group, with an average of 66.2+2.5 years ((51-70 years)); and there were 14 cases of traffic accident injury, 22 cases of falling injury and 14 cases of sprain. Patients in both groups were confirmed to be fresh closed fractures. Before admission, all patients received anteroposterior and lateral imaging of the ankle in Mindong Hospital Affiliated to Fujian Medical University, and the position and deviation of the fracture were determined by plain CT scan and three-dimensional reconstruction of the ankle. The study was approved by the Institutional Ethics Committee of Mindong Hospital Affiliated to Fujian Medical University (No.: 2022010; Date: February 12, 2022). All the selected participants were informed and provided written informed consent.

Furthermore, fracture classification was performed according to the Lauge-Hansen method. In the control group, there were 23 cases of supination-external rotation Type-III, 14 cases of supination and external rotation Type-IV and 13 cases of pronation-external rotation Type-IV. In the experimental group, there were 22 cases of supination-external rotation Type-III, 16 cases of supination-external rotation Type-IV and 12 cases of pronation-external rotation Type-IV. There was no significant difference in general data such as age, gender and etiology between the two groups (P>0.05), indicating the existence of comparability between groups. This study was approved by the Ethics Committee of our hospital.

### Inclusion criteria:

- Patients over 50 years old with fresh ankle fracture;
- Patients without symptoms of vascular and nerve injury before operation;
- Patients who had no wound infection before operation and whose fracture conditions allowed internal fixation;
- Patients who could tolerate surgery.

### Exclusion criteria:

- Patients with open ankle fractures;
- Patients with comminuted ankle fractures who cannot be fixed with screws;
- Patients with multiple fractures of both legs;
- Patients who could not follow the doctor’s advice for rehabilitation treatment after operation;
- Patients with diabetes, osteoporosis and malignant tumors.

Prior to the treatment, the enrolled patients were treated by manual reduction, followed by surgical treatment after the disappearance of the swelling of ankle soft tissue and blisters. Smoking and alcohol were forbidden in both groups during the period of treatment, accompanied by strict control of the diet, work and rest, and medication. Except for the difference in surgical fixation, patients in both groups shared similarities in the remaining treatment and nursing measures. Patients were not allowed to have out-of-bed activity without the permission of the medical staff postoperatively to avoid injury to the surgical site.

Patients in the control group received open reduction and internal fixation. Patients were adjusted to their supine position. After continuous epidural anesthesia, an incision of about 11 cm in length was made at the posterior edge of the lateral ankle fibula. The next step was tissue dissociation to expose the fracture of fibula and lateral malleolus, followed by the dissociation of peroneal tendon, anterior and posterior ligaments and avoiding the injury of tibiofibular ligament below external malleolus.

After reduction and fixation of posterior, lateral and medial malleolus successively, the posterior malleolus was fixed from the anterior and lateral directions to the posterior and lateral sides with cannulated lag screws. With full exposure of the lateral malleolus and distal fibula, after cleaning the hematoma and soft tissue around the fracture, anatomical plate of the distal fibula was used to fix the lateral malleolus. After that, an arc incision of medial malleolus (five cm in length) was made to fully expose the fracture focus of medial malleolus. Then, the medial malleolus was fixed with point-type reduction forceps and cannulated lag screws. The surgical reduction was reviewed by C-arm fluoroscopy X-ray machine. Finally, the incision was sutured and dressed after cleaning using hydrogen peroxide and indwelling the drainage catheter.

Patients in the experimental group received surgical treatment with fixation using buttress plate. The preoperative preparation and anesthesia methods were the same as those of the control group. The fixation sequence was lateral, posterior and medial malleolus. The patients were in their prone position. An incision of about 10 cm was made from the fibula to the posterior area, during which attention was paid to avoid injury of small saphenous vein and sural nerve. Tissues were dissociated until the exposure of flexor hallucis longus. The fracture of the lateral malleolus was exposed firstly through the anterior edge of the peroneal tendon, followed by reduction and fixation of the lateral malleolus using anatomical plate of the distal fibula.

Afterwards, from the inside to the outside, the fracture site of the posterior malleolus was entered through the space between the flexor hallucis longus and peroneus brevis muscle, followed by reduction of the fracture fragments of the posterior malleolus under direct vision. After the Kirschner wire was used temporarily for fixation from the posterior to the anterior region, the C-arm machine was applied to observe whether the articular surface was flat. For small fractures, cannulated screws were used for fixation, while for large fractures, the posterior ankle was fixed after shaping with buttress plate. An arc incision of medial malleolus (five cm in length) was made for reduction and fixation of medial malleolus with buttress plate. The length of the screw was controlled to ensure the smoothness of the joint.

The C-arm fluoroscopy X-ray machine was used to review the surgical reduction. The subsequent operation was the same as that of the control group. Patients in both groups received basic analgesia and anti-infection treatment after operation, and were provided with compression dressing using elastic band postoperatively. No random movement was allowed two weeks after operation. After four weeks, the plaster was removed and the removable brace was used continuously to protect the ankle for two weeks. During the wearing period of the removable brace, patients were guided to carry out no-weight-bearing full-range of motion training. Eight weeks after operation, patients were informed to carry out limited-weight-bearing full-range of motion training. The weight was adjusted according to the recovery of patients 10 weeks after operation. These patients were followed up for 12 months postoperatively.

### Outcome measures:

Quality of life: The questionnaire survey was conducted according to the comprehensive quality of life assessment questionnaire (GQOLI-74; 74 items in total), including physical function, mental function, social function and material life status. Patients with higher scores might have better quality of life.

### Ankle function:

One year after operation was evaluated by using Baird-Jackson score. The score was divided into multiple grades as follows: (1) excellent: 96-100; (2) good: 91-95; (3) moderate: 81-90; and (4) poor: 0-80. Good rate = one- number of subjects with poor ankle function. The postoperative complications were recorded for the performance of corresponding treatment.

### Statistical analysis:

All data were analyzed by SPSS19.0 statistical software. The counting data were expressed as the number of cases (percentage), and chi-square test was used for statistical analysis. While the measurement data were presented in the form of means±standard deviation () and analyzed by t-test. The ranked data were analyzed by Ridit-test. P<0.05 meant that the difference was statistically significant.

## RESULTS

According to the evaluation using questionnaire, the quality of life scores of the patients in the experimental group were higher than those in the control group in the aspects of physical function, mental function, social function, material life status, etc.(P<0.05). Compared with the control group, the Baird-Jackson score of the experimental group was improved after treatment, and the difference was statistically significant (P<0.05).Table-III

**Table-I:** Comparison of general data between the two groups

Groups	Age (years)	Traffic accident (n)	Falling from height (n)	Sprain (n)
Experimental group (n=50)	65.9+2.1	18 (36%)	19 (38%	13 (26%)
Control group (n=50)	66.2+2.5	14 (28%)	22 (44%)	14 (28%)
T/*χ*^2^ value	0..649	0.271	0.122	0.006
*P* value	0.517	0.602	0.762	0.933

**Table-II:** Comparison of quality of life between the two groups (*χ̅*±*s*)

Groups	n	Physical function	Mental function	Social function	Material life status
Experimental group	50	32.9±2.9	31.2±2.9	34.9±3.2	24.9±2.9
Control group	50	39.1±3.1	38.9±3.3	38.2±2.9	29.2±2.8
*χ*^2^ value		10.328	12.394	5.403	7.543
*P* value		0.000	0.000	0.000	0.000

**Table-III:** Comparison of Baird-Jackson quality between the two groups (*χ̅*±*s*)

Groups	n	Excellent	Good	Moderate	Poor	Good rate
Experimental group	50	27	15	7	1	49 (98.00)
Control group	50	21	10	14	7	43 (86.00)
U/*χ*^2^ value		8.547				4.891
*P* value		0.036				0.027

In the control group, there were four cases of postoperative incision infection and three cases of the breakage of internal fixation. In the experimental group, there were one case of incision infection and four cases of the breakage of internal fixation. The prognosis was good after timely treatment. There was no significant difference in the complications between the two groups (*P*>0.0; Table-IV).

**Table-IV:** Comparison of complications between the two groups (*n,* %)

Groups	n	Postoperative infection	Breakage of fixation	Total
Experimental group	50	4 (8.00)	3 (6.00)	7 (14.00)
Control group	50	1 (2.00)	4 (8.00)	5 (10.00)
*χ*^2^ value	-	-	-	0.757
*P* value	-	-	-	0.384

## DISCUSSION

This study compared the efficacy of a brace plate with conventional internal fixation in the treatment of ankle fractures. And it turns out, internal fixation with buttress plate has obvious advantages in the treatment of ankle fractures, which can effectively improve the quality of life and promote the rapid healing of fractures. Ankle fractures are generally caused by indirect violence, and are characterized by diverse types that are generally divided according to the direction and size of the force as well as position of the foot.^7^-^8^ In case of incorrect reduction of posterior malleolus fracture, or poor articular surface of the distal tibia, there may be high risk of postoperative complications such as traumatic arthritis, which seriously affects the quality of life of patients.^9^,^10^ The fragments of posterior malleolus fracture occur usually at the posterolateral side of the lower tibia. Most of them can recover voluntarily under the action of the distal posterior tibiofibular ligament after reduction and fixation.

While there were exceptions of no successful closed reduction, which require surgical treatment.^11^-^13^ Open reduction-internal fixation and internal fixation with buttress plate are currently used in the treatment of ankle fracture.^14^,^15^ Open reduction-internal fixation is somewhat effective clinically^16^,^17^, which, however, has some disadvantages, such as large incision, poor appearance, wound infection, exposure of internal fixation, etc. On the other hand, internal fixation with buttress plate is a novel therapeutic method. It has been reported that cannulated screw exhibits a satisfied effect on the biomechanical strength of posterior ankle fixation, which can play an anatomical reduction effect.

In this study, patients in the experimental group showed improved Baird-Jackson score after treatment, suggesting that the internal fixation with buttress plate is minimally invasive for the patients with knee fracture. Compared with the traditional open reduction treatment, the former method had shortened postoperative fracture healing time, more ideal clinical effect, and more satisfied long-term knee function recovery and range of motion.^18^ Based on the imaging findings of reduction and bone healing as well as the Baird-Jackson score, Zhou et al. concluded that the posterolateral approach of compression plate hollow screw internal fixation is an effective method for reduction and fixation of large posterior malleolus fractures.^19^ This is consistent with the conclusion of this study.

The postoperative ankle function recovery determined the quality of life of patients, which was the significance of the operation. Our study discovered that the quality of life score of patients in the experimental group was significantly higher than that of the control group. Findings in our study suggest that the internal fixation with buttress plate is beneficial to alleviate the pain, improve joint function and improve the quality of life of patients. Zhang et al.^20^ evaluated that plate fixation could achieve good clinical results from the aspects of ankle function, surgical pain and ankle motion, which was similar to the conclusion of our study. The conclusion of this study added clinical reference for the choice of treatment for such patients.

### Limitations

The number of subjects included in this study was limited, so the conclusions drawn may not be very convincing. In addition, we only analyzed and discussed the cases included in our hospital, which may not be representative enough. We look forward to a multi-center study in the future to reach more comprehensive conclusions.

## CONCLUSION

Internal fixation with buttress plate exhibits significant advantages in the treatment of ankle fractures, which can effectively improve the quality of life and promote the rapid healing of fractures. This approach is worthy of clinical promotion and application.

### Authors’ Contributions:

**ZL** **and** **QL:** Designed this study, prepared this manuscript, are responsible and accountable for the accuracy and integrity of the work.

**DG and YC:** Collected and analyzed clinical data.

**LG and** **KR:** Data analysis, Significantly revised this manuscript.
